# The efficacy outcomes in non-small cell lung cancer patients treated with PD axis inhibitor agents - a population-based study of the Vojvodina region

**DOI:** 10.3389/pore.2024.1611717

**Published:** 2024-07-12

**Authors:** Nensi Lalić, Marko Bojović, Daliborka Bursać, Darijo Bokan, Vesna Čeriman Krstić, Ivan Kuhajda, Biljana Parapid, Sanja Tomić, Aleksandar Šipka

**Affiliations:** ^1^ Faculty of Medicine, University of Novi Sad, Novi Sad, Serbia; ^2^ Institute for Pulmonary Diseases of Vojvodina, Sremska Kamenica, Serbia; ^3^ Oncology Institute of Vojvodina, Sremska Kamenica, Serbia; ^4^ Faculty of Medicine, University of Belgrade, Belgrade, Serbia; ^5^ University Clinical Center of Serbia, Belgrade, Serbia

**Keywords:** lung carcinoma, immune checkpoint inhibitors, immunotherapy, PD-L1 receptor, pembrolizumab

## Abstract

**Background:** By 2021, the FDA approved the use of the drugs pembrolizumab and atezolizumab in the first-line treatment of patients with high positivity of programmed death ligand-1 (PD-L1) in locally advanced and metastatic non-small-cell-lung cancer (NSCLC). This approval was the result of statistically significant evidence from international, multicentric clinical studies that all reported increasing progression-free survival (PFS) and overall survival (OS) in these patients.

**Methods:** In our study, we reported the demographic and clinical characteristics of 79 patients diagnosed with NSCLC with expression of PD-L1 ≥50% from January 2019 to December 2022 at the Institute for Pulmonary Diseases of Vojvodina, who received pembrolizumab therapy as the first-line treatment. Patients were divided according to the histological type of lung cancer as adenocarcinoma (ADC) or squamous cell carcinoma (SCC) of the lung. In 52 of the 79 patients, PFS and in 32 of them overall survival (censored OS) was shown according to the histological type of tumor, the tumor proportion score (TPS) of PDL-1 expression, and the metastatic status within the Tumor Nodes Metastasis (TNM) disease classification. Independent factors of death outcome were shown by multivariable proportional hazard regression analysis.

**Results:** The study included 79 patients diagnosed with NSCLC with an expression of PD-L1 ≥50%, 50 (63.3%) patients with ADC, and 29 (36.7%) patients with SCC, whose 55 (69.6%) PDL-1 expression was obtained from broncho biopsy (BB). The majority of patients, 49 (62%), had a TPS PD-L1 score of 51%–79%. Median, PFS for adenocarcinoma was 22 months and censored OS was 27 months, while for squamous cell carcinoma, median PFS was 12 months, and censored OS was 21 months. M1b disease stage, which was the most common in patients, had a PFS of 16 months and a censored OS of 18 months.

**Conclusion:** Pembrolizumab monotherapy in patients with NSCLC in the fourth stage of the disease and with the positivity of the immune checkpoint protein TPS PD-L1 above 50% represents a safe therapy that allows a satisfactory period without disease progression and overall survival with acceptable treatment complications.

## Introduction

The survival of patients with lung cancer in the last decade has had an increasing trend if data from all over the world is compared. In particular, NSCLC accounts for about 85% of all malignant lung tumors, with ADC (40%) taking the lead to SCC and other pathological types of lung cancer [1]. Targeted molecular therapy has significantly improved the treatment of advanced and metastatic NSCLC when compared to standard chemotherapy. Among the new treatments for advanced lung cancer now available, immunotherapy is the newest and most promising [2]. Malignant tumor cells have characteristics that allow them to spread and maintain their neoplastic state, evade the immune system, and inhibit the immune response, by preventing the activation of immune T cells increasing the expression of immune checkpoint proteins on T cells [3]. The basis of new therapeutic procedures was the inhibition of those pathways that led to the evasion of the immune system through immune checkpoints, which changed the paradigm of cancer treatment. The drugs of this group are called immune checkpoint inhibitors (ICIs). The suppression of signaling pathways involving programmed cell death ligand-1 (PD-L1) and programmed cell death-1 (PD1) increases the functionality and the response strength of CD8⁺ T (cluster of differentiation 8 thymus) cells. By suppressing the signaling that PD-1 receives from its ligand, it is possible to restore the activity of T lymphocytes and increase their antitumor activity with a further increase in PD-L1 expression in the tumor microenvironment (TME) [4]. Immunotherapy in oncology began with the approval of antibody therapy against cytotoxic T lymphocyte antigen-4 (CTLA-4) in 2011 for the treatment of metastatic melanoma. This was followed by the approval of seven additional immune checkpoint inhibitors (ICIs) focusing on suppressing the PD-1 pathway: ipilimumab, pembrolizumab, nivolumab, atezolizumab, avelumab, durvalumab, and cemiplimab, which are currently used to treat 18 different types of cancer [5]. There is evidence that PD-L1 is found in a higher percentage in NSCLC and it is expressed in the majority of tumor-infiltrating lymphocytes, which has been the reason for to introduce PD-L1 or PD-1 inhibitors into the treatment of NSCLC. The level of PD-L1 expression in the tumor shows a better response and longer survival rate (proportional score >50% of tumor cells for first-line immunotherapy and >1 for second-line immunotherapy) compared to conventional chemotherapy. Based on these results, the FDA approved the drug pembrolizumab in the first-line therapy of advanced NSCLC for ≥50% PD-L1 expression without the presence of epidermal growth factor receptor (EGFR) mutation and anaplastic lymphoma kinase gene (ALK) rearrangement [6]. The KEYNOTE 024 study investigated the role of pembrolizumab in first-line therapy in patients with high PD-L1 expression (≥50%). Updated analyses showed that pembrolizumab as monotherapy improved overall survival (OS) compared with platinum-based chemotherapy, with a median overall survival (median OS) of 30.0 vs. 14.2 months [7]. It was also proven that there was a synergistic effect of the drug pembrolizumab with chemotherapy, that both agents reduced the activity of T regulatory cells and increased the presentation of tumor antigen cells [8]. The third phase KEYNOTE 189 study primarily studied and confirmed improvements in PFS and OS in the group of patients with combined therapy of pembrolizumab and chemotherapy [9]. The KEYNOTE 407 study related to combined immunochemotherapy in squamous metastatic NSCLC showed an improvement in PFS and OS but also the effect on PD-L1 expression [10, 11]. This trial changed the indication for receiving immunotherapy for patients with advanced squamous NSCLC, which until recently was treated with cytotoxic chemotherapy due to the absence of targetable aberrations in this pathohistological type of tumor [12]. The purpose of this paper is to elaborate on the demographic and clinical characteristics of metastatic NSCLC patients diagnosed as PD-L1 positive (with over 50% positivity) who underwent first-line therapy with pembrolizumab and to show disparities between squamous and non-squamous NSCLC, segmented by the number (1, 2, 3, or more) and location of metastases (liver, bone, and brain, and other sites).

## Materials and methods

### Methodology

The retrospective study involved 79 patients with metastatic ADC and SCC lung cancer who were treated with pembrolizumab as the first line of treatment from January 2019 to December 2022 at the Institute for Pulmonary Diseases of Vojvodina. Out of these 79 patients, 52 who started the treatment by the end of 2021 were grouped together in a subgroup of the study so that the PFS and censored OS outcomes could be observed over a 2-year period. Inclusion criteria: patients above 18 years old, pato-histological confirmed NSCLC, metastatic disease, previously not treated with chemo or radiotherapy. Exclusion criteria: low ECOG performance status, another synchronic malignancy, heavy comorbidities. The minimum follow-up period was 24 months.

### Immunohistochemical analysis

After sectioning Formalin-Fixed-Paraffin-Embedded (FFPE) on 4–5 µm of tumor tissue be mounted on Fisherbrand Superfrost Plus slides and stained using the PD-L1 clone 22C3 pharmDx kit and Dako Automated Link 48 platform (Agilent Technologies/Dako, Carpinteria, CA, United States).

The Tumor Proportion Score (TPS) was calculated as the percentage of at least 100 viable tumor cells. Based on partial or complete staining of the membrane all samples were divided into one of three groups: with <1% (no expression), 1%–49% (low expression), or ≥50% (high expression) positive tumor cells.

### Statistical analysis

Attribute descriptors were presented of frequency and percentage. The difference in frequency of attribute descriptors was tested using the Chi-Square test. The normality of data distribution was assessed using the Shapiro-Wilk test. The values of PFS and censored OS were depicted using the median, Kaplan-Meier curves, and Cox regression analysis.

A statistical significance level of *p* < 0.05 was employed. Results were displayed both in tabular format and using Kaplan-Meier curves. Tables were created using Microsoft Word v 2021. IBM SPSS Statistics v26 and JASP v0.11.3 were utilized for statistical data analysis.

### Ethical statement

This study was approved by the Institutional Ethical Committee (Ethical Committee of the Institution for Pulmonary Diseases of Vojvodina No. A203/2). The identities of all the patients from our study was removed and the study was conducted according to the Helsinki Declaration of the World seventh revision.

## Results

We presented 79 patients with metastatic NSCLC who received monotherapy with the drug pembrolizumab. Demographic and clinical characteristics of the patients according to age, gender, smoking status, and comorbidities are shown in Table 1.

**TABLE 1:** Demographic and clinical characteristics of the patients treated with pembrolizumab from January 2019 to December 2022.

		N = 79
Age (years)		64.9 (±6.71)
Gender	Males	48 (60.8%)
	Females	31 (39.2%)
Smoking status	Never-smoker	7 (8.9%)
	Former smoker	61 (77.2%)
	Current-smoker	11 (13.9%)
Comorbidities	COPD	10 (12.7%)
	Cardiovascular diseases	23 (29.1%)
	Renal diseases	2 (2.5%)
	Diabetes Mellitus	1 (1.3%)
	Rheumatoid arthritis	1 (1.3%)
	Autoimmune diseases	1 (1.3%)
	Other pulmonary diseases	2 (2.5%)
	Other diseases	3 (3.8%)

For all 79 patients in the study who received pembrolizumab in the first line of treatment, ADC or SCC lung cancer in the metastatic stage of the disease was previously confirmed histologically or from a cellblock. The majority, i.e., 50 of them (64.1%), had lung ADC. The tumor tissue sample most often obtained for the PD-L1 test was a biopsy from bronchoscopy, among 55 (69.6%) patients. The number of CT-guided core biopsies is low in our study, we rarely performed that procedure. These results are shown in Table 2.

**TABLE 2:** Tumor characteristic; sample types; sampling method.

Hystological type	Adenocarcinoma	50 (63.3%)
	Squamous	29 (36.7%)
Type of samples	Histology	77 (97.5%)
	Cytology (Cytoblock)	2 (2.5%)
Type of sampling	Bronchoscopy	55 (69.6%)
	Surgery	13 (16.5%)
	Tru-cut	2 (2.5%)
	Biopsy of metastasis	5 (6.3%)
	Cytoblock from bronchoscopy	2 (2.5%)
	Cytoblock from pleural effusion	2 (2.5%)

Given that among our patients, immunotherapy was applied only to patients in the metastatic stage of the disease, we were interested in the representation of tumor metastases according to the number and localization in each patient (Table 3). Most of the patients were in the M1b stage of the disease, and the most common localization of metastases was pulmonary metastasis, in 28 (36.7%) patients.

**TABLE 3:** Metastasis characteristics.

TNM M status	M1a	29 (36.7%)
	M1b	41 (51.9%)
	M1c	9 (11.4%)
Localization of metastasis	Lung	28 (36.7%)
	Brain	7 (8.9%)
	Liver	6 (7.6%)
	Bone	11 (13.9%)
	Adrenal node	9 (11.4%)
	Pleura	1 (1.3%)
	Multi	9 (11.4%)
	Other	8 (10.1%)

PD-L1 protein expression in tumor cells and tumor-infiltrating immune cells (ICs) were assessed by immunohistochemistry. The tumor proportion score (TPS) as the percentage of viable tumor cells determined the PD-L1 protein expression in the tumor. All samples from the patients in the study had TPS as ≥50% (high expression) positive tumor cells of the PD-L1 test. The patients were divided into three subgroups: PD-L1 with 50% positivity, PD-L1 with 51%–79% positivity, and PD-L1 over 80% positivity evaluation. The positivity of the test was also compared according to the histological type of the tumor and according to the metastatic status of the tumor. PD-L1 positivity of 51%–79% was most common in both histological types of tumors and within M1b metastatic tumor status. There was no statistically significant difference in the strength of PD-L1 expression for either of the mentioned tumor characteristics (Table 4).

**TABLE 4:** TPS PD-L1 by histology type and by M status.

		PD L1 status			*p*-value
		50%	51%–79%	80% and more	
Histological type	ADC	2 (4.0%)	28 (56.0%)	20 (40.0%)	0.127^a^
	SCC	2 (7.1%)	21 (75.0%)	5 (17.9%)	
Characteristics of metastasis	M1a	2 (6.9%)	17 (58.6%)	10 (34.5%)	0.509^a^
	M1b	2 (4.9%)	28 (68.3%)	11 (26.8%)	
	M1c	0 (0%)	4 (44.4%)	5 (55.6%)	

ADC, Adenocarcinoma; SCC, Squamous cell carcinoma; PD L1, Programmed cell death receptor ligand 1.

^a^ Chi-Square test.

In this paper, we monitored the first follow-up response (FUP1) according to the modified Response Evaluation Criteria in Solid Tumors (RECIST version 1.1) used in malignant tumors immunotherapy trials, so-called iRECIT, for all 79 patients who received pembrolizumab therapy for at least 3 months until the end of 2022. Therapeutic response was presented as partial regression (PR), stable disease (SD), progressive disease (PD), and pseudoprogression disease (psPD). There was no statistically significant difference at FUP1 according to the histological tumor type, i.e., at FUP1 most patients of both histology types of tumors had stable disease (SD). The same applied to M status, that is, patients of M1a, M1b, and M1c status most often had SD at FUP1 (Table 5).

**TABLE 5:** Response (FUP1) by histological type of tumor and by M status.

		Response				*p*-value
		PR	SD	PD	psPD	
Histological type	ADC	16 (32.0%)	28 (56.0%)	2 (4.0%)	3 (6.0%)	0.136^a^
	SCC	5 (17.9%)	17 (60.7%)	3 (10.7%)	0 (0%)	
Characteristics of metastasis	M1a	9 (31.0%)	16 (55.2%)	0 (0%)	2 (6.9)	0.363^a^
	M1b	10 (24.4%)	26 (63.4%)	4 (9.8%)	0 (0%)	
	M1c	2 (22.2%)	4 (44.4%)	1 (11.1)	1 (11.1%)	

ADC, Adenocarcinoma; SCC, Squamous cell carcinoma; PR, Partial Response; SD, Stable Disease; PD, Progressive Disease; psPD, Pseudoprogression Disease.

^a^ Chi-Square test.

We also investigated the type of disease progression according to the histological type of tumor and according to the metastatic stage of the disease, whereby the disease progression causes were marked as locoregional disease progression or distant (appearance of new metastatic lesions). The most common type of progression was the occurrence of distant progression in both ADC and SCC lung cancer, as well as in previous (before therapy) existing M1b disease stage. According to different histology or different M statuses, there was no statistically significant difference in the type of disease progression (Table 6).

**TABLE 6:** Progression causes by histology type and by M status.

		Progression			*p*-value
		Locoregional	Distant	Without progression	
Histological type	ADC	5 (10.2%)	11 (22.4%)	33 (67.3%)	0.953^a^
	SCC	3 (12.0%)	5 (20.0%)	17 (68.9%)	
Characteristics of metastasis	M1a	2 (7.4%)	5 (18.5%)	20 (74.1%)	0.608^a^
	M1b	4 (10.0%)	10 (25.0%)	26 (65.0%)	
	M1c	2 (25.0%)	1 (12.5%)	5 (62.5%)	

ADC, Adenocarcinoma; SCC, Squamous cell carcinoma.

^a^ Chi Square test.

We also examined the reasons for discontinuation of pembrolizumab therapy (disease progression, treatment complications occurrence, worsening of the patient’s ECOG performance status, death). There was no statistically significant difference in the reasons for discontinuation of therapy according to histology type of tumor or M status of the disease (Table 7).

**TABLE 7:** Therapy discontinuation caused by histology type and by M status.

		Response					*p*-value
		Progression	Complications	ECOG	Death	Without disrupt	
Histological type	ADC	13 (30.6%)	6 (12.0%)	2 (4.0%)	6 (12.0%)	23 (46.0%)	0.363^a^
	SCC	6 (26.9%)	2 (7.7%)	3 (11.5%)	5 (19.2%)	10 (38.5%)	
Characteristics of metastasis	M1a	6 (22.2%)	2 (7.4%)	2 (7.4%)	2 (7.4%)	15 (55.6%)	0.666^a^
	M1b	12 (29.3%)	4 (9.8%)	2 (4.9%)	7 (17.1%)	16 (39.0%)	
	M1c	1 (11.1%)	2 (22.2%)	1 (11.1%)	2 (22.2%)	3 (33.3%)	

ADC, Adenocarcinoma; SCC, Squamous cell carcinoma; ECOG, Eastern Cooperative Oncology Group.

^a^ Chi Square test.

Therapy postponement (up to 30 days, over 30 days, without delay) as well as the cycle in which the therapy was delayed are shown in Table 8.

**TABLE 8:** Postponed therapy distribution.

		N = 79
Postpone	Up to 30 days	12 (15.2%)
	30 days and more	3 (3.8%)
	Without postponement	64 (81.0%)

After the discontinuation of pembrolizumab therapy, we examined the continuation of other types of therapy according to the histology type of the tumor and according to the M status of the disease. The most common type of continued therapy for all observed subgroups of patients was supportive therapy, followed by chemotherapy (five patients with ADC, three patients with SCC, four patients with M1a, and four patients with M1b status), radiotherapy was received by one patient with ADC and one patient with M1b disease status, while one patient with SCC and one patient with ADC received chemoradiotherapy.

The most common adverse event (AD) in all subjects was the occurrence of hypothyroidism, which did not lead to discontinuation, but only to therapy postponement up to 30 days. A specialist endocrinologist was involved in the treatment and monitoring of hypothyroidism. Therapy complications are shown in Table 9.

**TABLE 9:** Pembrolizumab therapy complications.

			N = 79
Complications	Yes	Hypothyroidism	11 (13.9%)
		Pneumonitis	5 (6.3%)
		Kidney disease	4 (5.1%)
		Infections	4 (5.1%)
		Pneumonia	3 (3.8%)
		Skin toxicities	3 (3.8%)
		Hepatic failure	3 (3.8%)
		Hematological toxicity	3 (3.8%)
		Pulmonary thromboembolism	2 (2.5%)
	No		41 (51.9%)

A group of patients who underwent surgical treatment of lung tumors before the appearance of metastatic disease was specially monitored. There was a total of 8 patients, of which before pembrolizumab therapy 3 had radiotherapy, chemotherapy 4 of them, and chemo and radiotherapy one of them. These patients, i.e., the therapeutic outcome with pembrolizumab, was not compared to patients who did not have surgery. Also, one patient had palliative radiotherapy for some metastases or palliative radiotherapy for superior vena cava syndrome (SVCS) before or during the administration of pembrolizumab. The treatment outcome among these patients compared to patients who did not have palliative radiotherapy while receiving the drug pembrolizumab was not separately compared.

### PFS and censored OS

**FIGURE 1:**
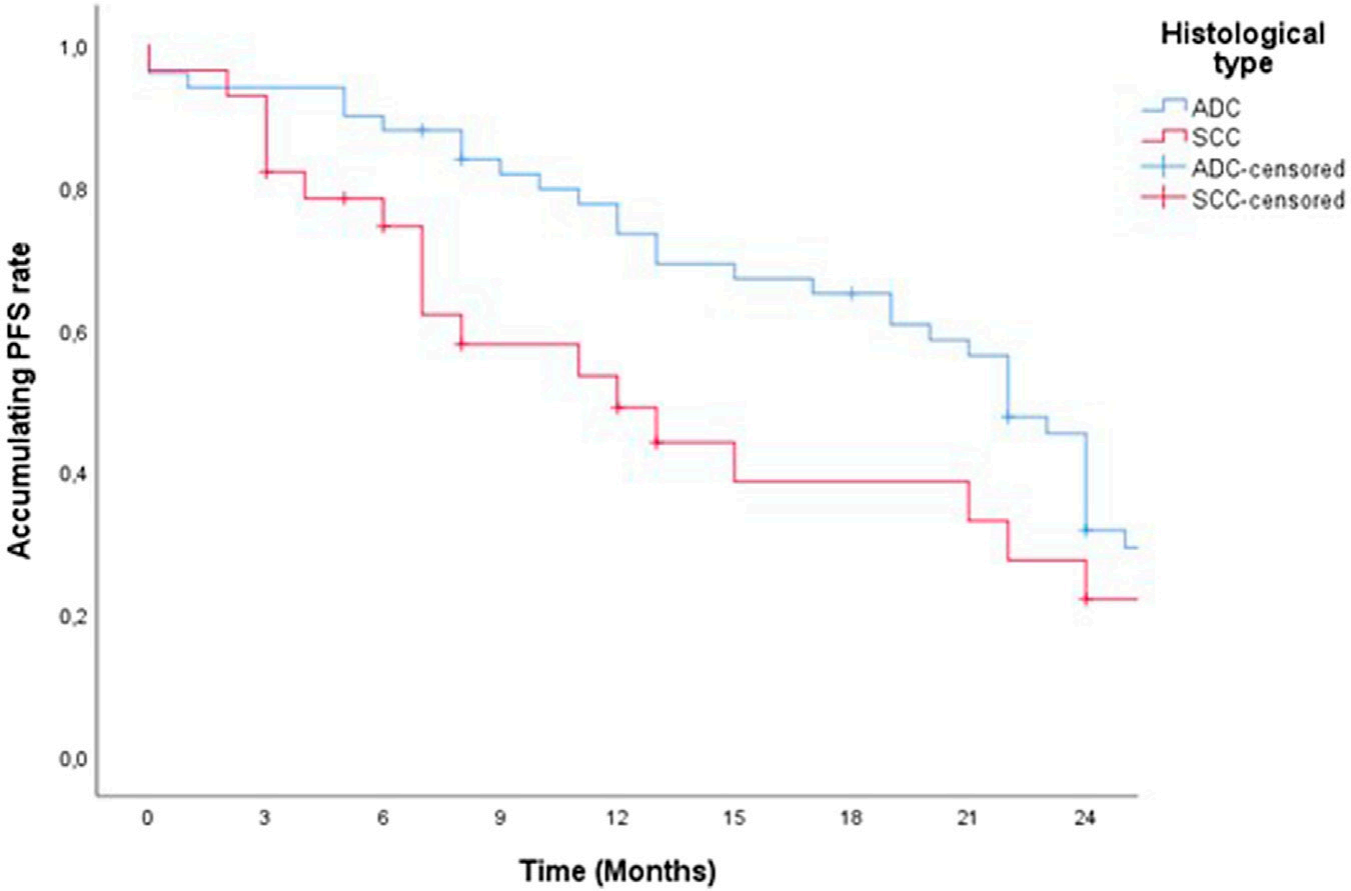
Two-year PFS of patients treated with pemrolizumab according to histological tumor type.

**FIGURE 2:**
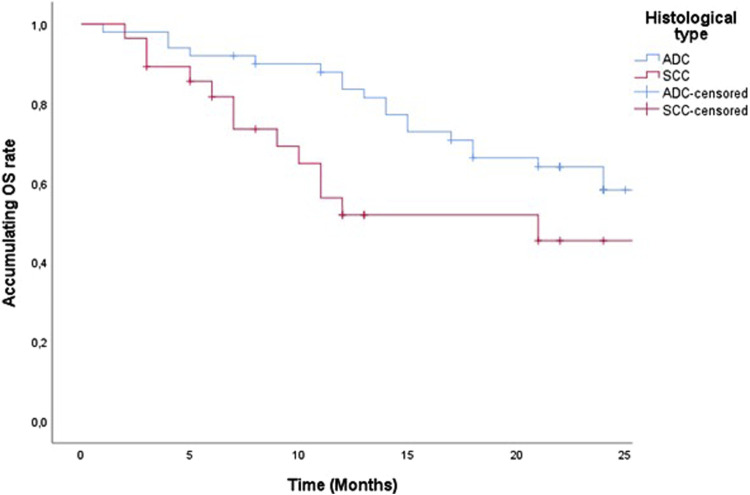
Two-year censored OS of patients treated with pemrolizumab according to histological tumor type.

79 patients in our study started therapy with the drug pembrolizumab from December 2019 to January 2022. For a 2-year PFS and censored OS presentation, we singled out 52 patients whose pembrolizumab therapy was scheduled to start by the end of 2021. Accordingly, some of the 52 patients were alive by the end of 2022, the OS value was lower than the PFS and was calculated and shown in our study as censored OS. PFS and censored OS were observed according to the histology type of the tumor, with a longer 2-year disease progression and overall survival among patients with ADC (Figures 1, 2). Median PFS (censored) for ADC was 22 months, and for SCC was 12 months (half of the observed patients with ADC have been progressed in 22 months).

Median OS (censured) for ADC was 27 months and for SCC 21 months (half of observed patients were dead in 27 months and 21 months).

**FIGURE 3:**
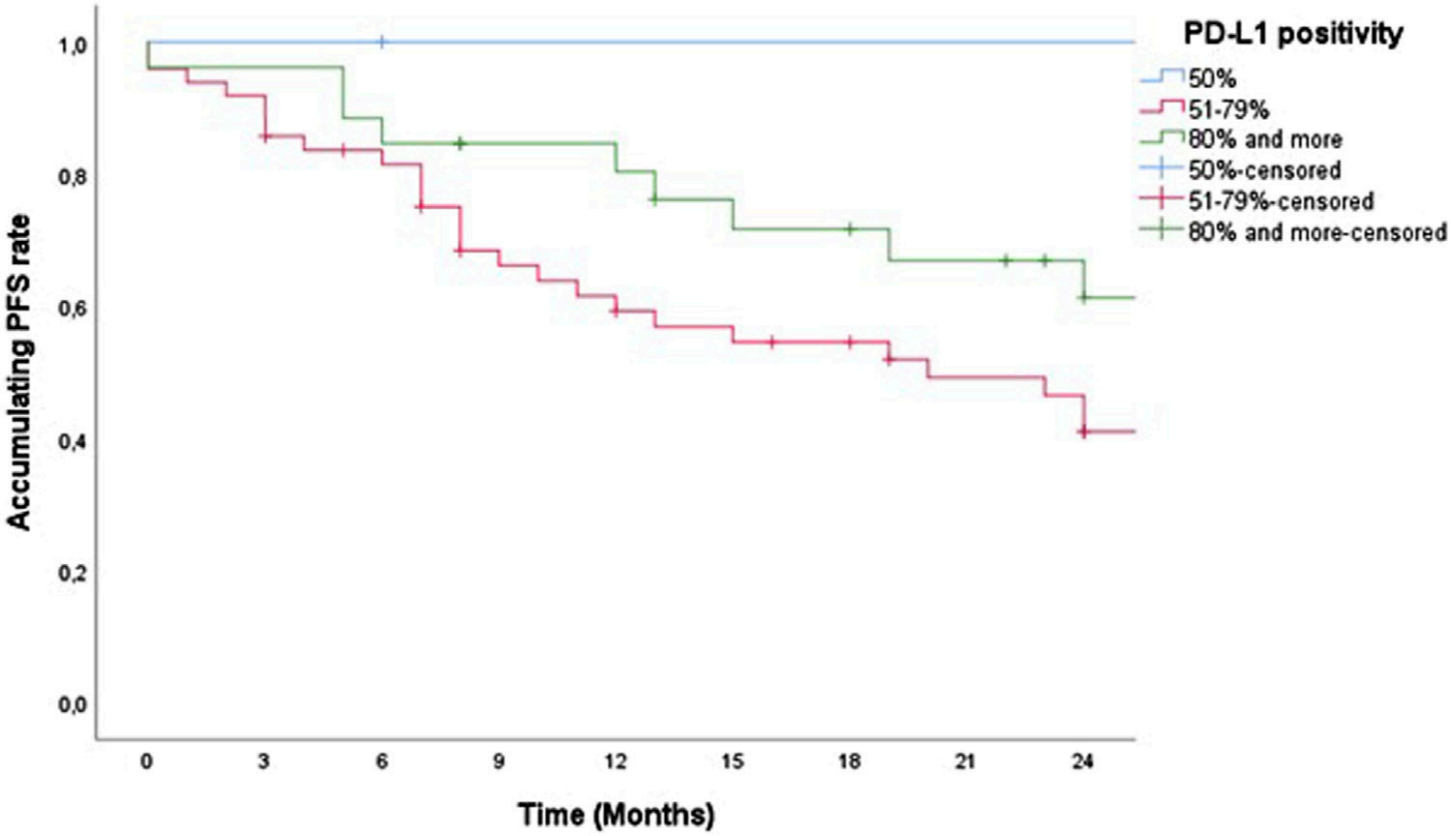
Two-year PFS of patients treated with pemrolizumab according to PD-L1 positivity.

**FIGURE 4:**
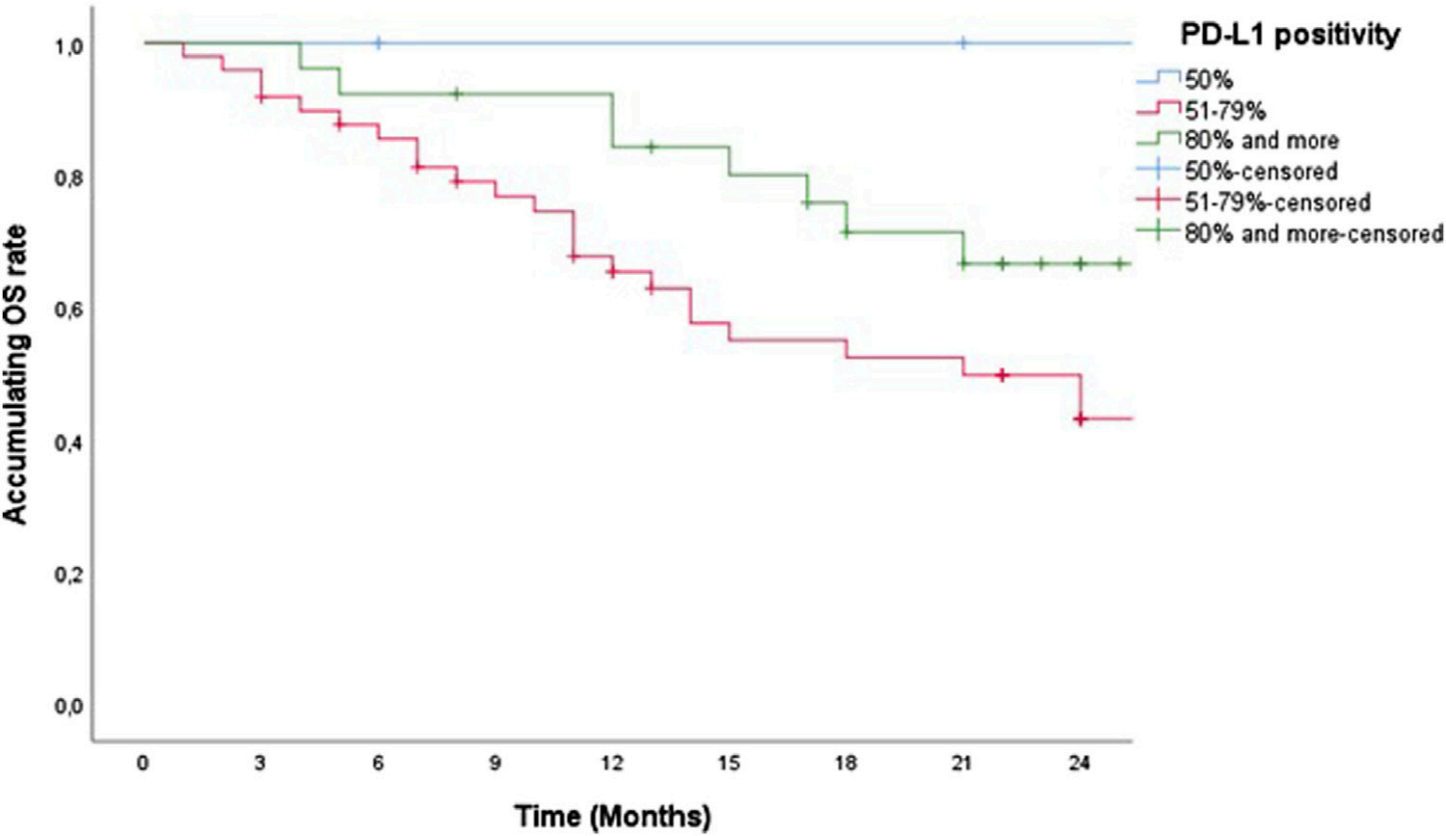
Two-year censored OS of patients treated with pemrolizumab according to PD-L1 positivity.

According to the percentage of PD-L1 positivity (PD-L1 50%, PD-L1 51%–79%, and PD-L1 over 80%) there was no statistically significant difference in 2-year PFS and censored OS (Figures 3, 4).

**FIGURE 5:**
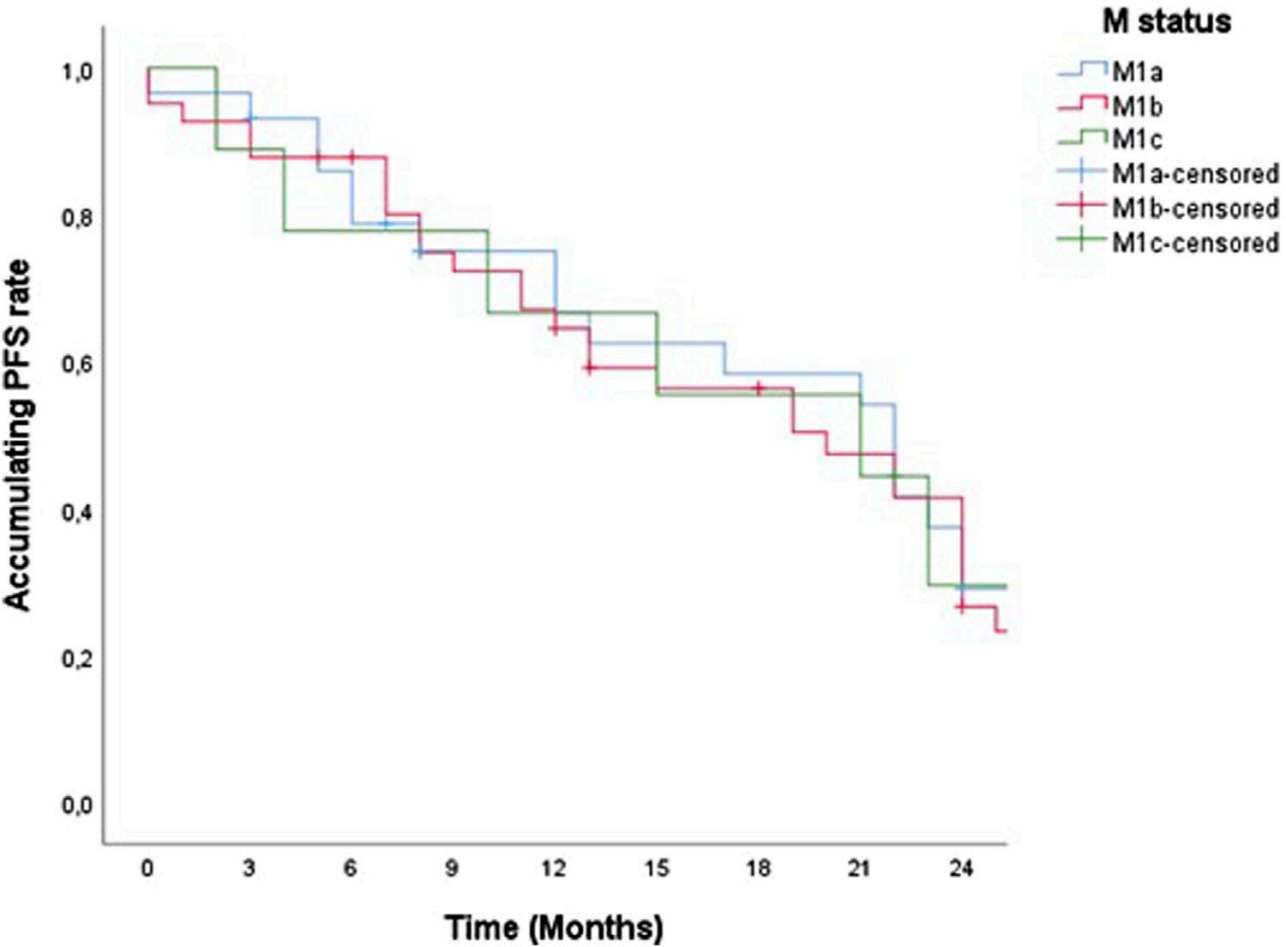
Two-year PFS of patients treated with pemrolizumab according to M status.

**FIGURE 6:**
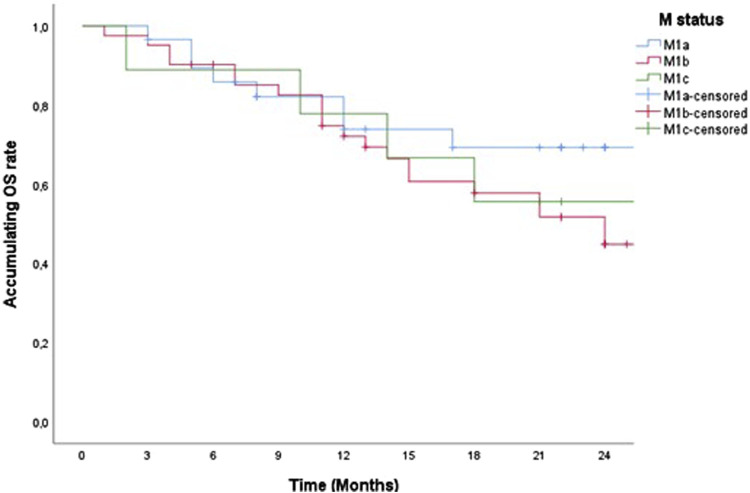
Two-year censored OS of patients treated with pemrolizumab according to M status.

According to M disease status (M1a, M1b, and M1c) there was no statistically significant difference in 2-year PFS and censored OS (Figures 5, 6).

## Discussion

Among various malignancies, NSCLC is a type of solid tumor that showed an extremely good therapeutic response to the introduction of immunotherapy in the last decade. For the prevention and regulation of malignant tumors, immunosurveillance is essential [13]. The activity of the immune system T-cells as the main active component of the immune response is regulated by stimulatory as well as inhibitory signals called checkpoints. The main checkpoint that regulates the immune response is the interaction between the PD-1 receptor and its programmed cell death ligand-1 (PD-L1). Monoclonal antibody therapy can effectively inhibit these proteins and thus modulate tumor growth and development [14]. Pembrolizumab (Keytruda^®^), formerly lambrolizumab, is a humanized IgG4 kappa monoclonal antibody that inhibits PD-1 receptor activity. The drug was originally approved by the Food Drug Administration (FDA) in 2014, and by the European Commission in 2015 for the treatment of unresectable and metastatic melanoma that progressed after ipilimumab therapy [15]. So far, there have been several trials where lung cancer patients have been treated with pembrolizumab. In KEYNOTE-010, pembrolizumab in the second line of treatment showed long-term benefits compared to chemotherapy (docetaxel), while the patients were previously treated with chemotherapy. According to PD-L1 status, patients were divided into subgroups of PD-L1 TPS (tumor proportion score) ≥50% and ≥1% [16]. A randomized, phase 2 KEYNOTE-021 study showed a significantly better therapeutic response among the patients treated with pembrolizumab and chemotherapy compared to chemotherapy alone. In this study, pembrolizumab was given as a first-line treatment in combination with chemotherapy to patients with advanced or metastatic NSCLC [17]. KEYNOTE-024 as a randomized phase 3 multicenter study investigated the therapeutic response of the first-line pembrolizumab as monotherapy versus first-line platinum-based chemotherapy among patients with NSCLC. All patients in the study with advanced or metastatic LC had PD-L1 TPS ≥50% [18]. KEYNOTE-042 is a multicenter phase 3 study similarly designed as KEYNOTE-024, which enrolled treatment-naïve patients whose tumors showed strong (PD-L1 TPS ≥50%) versus weak (PD-L1 TPS 1%–49%) PD-L1 positivity [19]. Both studies showed a better therapeutic response in patients treated with immunotherapy. In our study, we showed the effect of pembrolizumab treatment as monotherapy in the first-line treatment of advanced or metastatic NSCL with PD-L1 TPS ≥50%, where the primary objective of the study was to illustrate disparities and therapeutic response between metastatic squamous and non-squamous NSCLC, according to the number and location of metastases and according to PD-L1 TPS positivity.

Testing for PD-L1 expression is performed using a diagnostic immunohistochemical test. PD-L1 expression requires histological samples, as the sample formalin fixation should be done with at least 100 tumor cells [20]. Tissue sampling for PD-L1 testing in this study was most often from biopsies obtained by bronchoscopy, which corresponded to the patients included in the study, who all had metastatic disease. Only a small number of patients had a tissue sample resected during surgery, and these were the patients who after a certain time progressed to stage IV of the disease, and a tissue sample was taken from the initial phase of the disease. One of the studies evaluated the concordance of PD-L1 expression between bronchoscopic small biopsies and surgical resections. PD-L1 expression was investigated in 79 patients for whom bronchoscopic biopsy specimens and surgical resection specimens were available. The concordance of PD-L1 expression between small biopsies and surgical resections was 92.4% [21]. In our study, two samples for PD-L1 testing were from the cytoblock obtained from the endobronchial ultrasound transbronchial needle aspiration (EBUS-TBNA) bronchoscopy. In on other study the concordance rate between EBUS-TBNA versus surgical specimens was 87% for PD-L1 ≥1% and 82% for PD-L1 ≥50%. However, the sensitivity of EBUS-TBNA samples dropped from 72% to only 47% at a PD-L1 expression cutoff of ≥50% (vs. ≥1%), raising concerns about false-negative PD-L1 results on EBUS-TBNA samples [22].

In our study, patients were treated with pembrolizumab monotherapy at a dose of 200 mg of body weight every 21 days, where all patients had metastatic disease, PD-L1 TPS ≥50%, ECOG (European Cooperative Oncology Group) status 0 or 1. The smallest number of drug cycles received was 3, and the largest was 56. After 2 years of this type of therapeutic regimen, 6 patients switched to the regimen of administering the drug dose of 400 mg every 6 weeks. Currently, the definition of permanent response among immunotherapy patients has been controversial. Permanent response is defined as the patient’s progression-free survival (PFS) exceeding three times the median PFS of the same cohort [23]. Permanent responses to administered ICIs can last for months or years, with some patients even having improved responses to ICIs over time according to iRECIST criteria and thus even longer censored OS [24]. The National Comprehensive Cancer Network (NCCN) guidelines recommend that patients with NSCLC should receive maintenance ICIs for 2 years if they have received first-line immunotherapy. Maintenance therapy is administered for 2 years in patients who received first-line immunotherapy and until disease progression in patients who received second-line immunotherapy [25, 26]. In tumors, there may be a transient increase in the volume or number of lesions (temporary progression) after ICI treatment, but after that period partial regression (PR) or stable disease (SD) may occur, which is defined as pseudoprogression [27]. Pseudoprogression, as an unusual but useful pattern of treatment with ICIs, should be emphasized and carefully recognized. The increased tumor volume is likely to be the result of the recruitment of activated T cells to the tumor site during ICI treatment. Before these cells achieve their antitumor functions, they lead to inflammation and tumor volume enlargement, as well as immune infiltration, edema, and necrosis. The incidence of pseudoprogression varies among tumor types but is rarely >10% [28–31]. Pseudoprogression often occurs after initial treatment with ICIs [29]. In our study, at the first follow-up visit (FUP1), a total of six patients had psPD (pseudoprogression disease) response, and during the entire study, a total of 7 patients had psPD, as in the above-mentioned studies, at the beginning of treatment. All patients continued therapy, with PFS and censored OS being significantly longer compared to pseudoprogression-free patients if we observe a 2-year overall survival.

A total of 38 out of 79 (48%) patients had complications of pembrolizumab administration in our study, which is a good result compared to other studies where the frequency of complications in monotherapy with ICIs ranged from 15% to 90% [19, 32]. In a systematic review of 50 studies, the incidence of grade 3/4 adverse events (AEs) was 21% (range 0%–66%) [33]. Comparing our results with studies where immunotherapy was given as monotherapy for NSCLC, the most frequent AE was hypothyroidism CTCAE grade 1, classified according to Common Terminology Criteria for Adverse Events (CTCAE), found in 11 (13%) patients, compared to other complications that were individually represented by no more than 1 (1%). The postponement of therapy until the stabilization of the hormone concentration by medication did not amount to more than 30 days, and there was no interruption of therapy for that reason. In a retrospective cohort study of 1,246 patients treated with ICIs, immune-related AEs (IRAEs) occurred in 518 (42%) patients. Subclinical thyrotoxicosis (n = 234) was the most common thyroid IRAE, followed by overt thyrotoxicosis (n = 154), subclinical hypothyroidism (n = 61), and overt hypothyroidism (n = 39) (89). Thyroid dysfunction is the most common endocrine IRAE in NSCLC [34].

In this paper, we were able to show 2-year overall survival (censored OS) and progression-free survival (PFS) according to certain clinical and pathological tumor characteristics for 52 patients out of the total of 79 patients who received pembrolizumab therapy by the end of 2021. The studies which included pembrolizumab as monotherapy showed the following results: KEYNOTE-010 study showed better OS over docetaxel for both groups of PD-L1 TPS ≥50% and ≥1% positivity, (hazard ratio [HR] 0.53; 95% CI and HR, 0.69; 95% CI, *p* < 0.00001, respectively) [16]. In the KEYNOTE-021 Cohort G study, PFS for pembrolizumab in combination therapy (n = 60) versus chemotherapy (n = 63) was (median: 24.5 versus 9.9 months; hazard ratio: 0.54; 95% confidence interval: 0.35–0.83). Median overall survival was 34.5 versus 21.1 months (hazard ratio: 0.71; 95% confidence interval: 0.45–1.12) [35].

In our study, 59 (64.1%) patients had ADC, and 20 (35.9%) of them had squamous cell carcinoma (SCC). According to the histological type of lung cancer, patients with ADC had a longer censored PFS and censored OS compared to SCC, which amounted to (PFS HR 0.626 95% CI 0.358–1.09, *p* = 0.101 and censored OS HR, 0.550; 95% CI 0.280–1.08, *p* = 0.08). A multicentric study published in 2022 reported potential differences in the efficacy of ICIs in the treatment of squamous NSCLC and non-squamous NSCLC. ICI monotherapy improves OS in SCC and ADC (OS HR 0.71, 0.80), and overall all of the studies showed a greater OS for SCC (OS HR 0.89, 95% CI 0.80–0.99). For PFS ICI monotherapy also showed a reduction in the risk of disease progression and even more so for SCC compared to ADC (35% for SCC vs. 10% for ADC) [36].

We observed both PFS and censored OS in our study according to the degree of PD-L1 positivity, whereby we divided patients into three groups (PD-L1 TPS = 50%, 51%–79%, and over 80%). There was no statistically significant difference in PFS and censored OS according to the degree of high positivity of PD-L1 TPS (PFS, *p* = 0.587 and censored OS, *p* = 0.583) because all the groups that were observed for this characteristic are essentially groups with high expression of PD-L1. The correlation analysis between PD-L1 expression levels and clinicopathological parameters was shown in 1,008 SCC or ADC, it was concluded that PD-L1 expression is higher in men, smokers, squamous cell carcinoma tumors with a maximum diameter >3 cm, patients with poor differentiation and/or high TNM disease stage. Among patients with well to moderately differentiated ADC, clinical stages I-II, without a history of smoking, lymph node metastases, or distant metastases, no PD-L1 testing was recommended. However, a strong recommendation for testing has been established for patients with SCC who have lymph node metastases or poorly differentiated NSCLC with N3 disease, especially smokers with N3 disease [37, 38].

According to the localization of metastases and their number and taking into account the TNM classification for the M stage of the disease patients in our study were divided into M1a, M1b, and M1C stages. The M1c rate was so low in the study maybe because we did not perform PET/CT on all the patients, only to some of them. In addition, there was no difference in PFS and censored OS between these three groups of patients with NSCLC treated with pembrolizumab (PFS, *p* = 0.473 and censored OS *p* = 0.09). So far, comparative studies that would monitor PFS and censored OS according to this characteristic have not been done, but in one of the multicentric studies that included 16 randomized clinical trials (RCTs) and 14 observational trials, a survival improvement for ICIs (ICI monotherapy, ICI + chemotherapy, dual ICI therapy and dual ICI + Chemotherapy) versus standard therapies was reported among NSCLC patients with liver metastases: (PFS HR, 0.77; 95% CI, 0.61–0.97; censored OS HR, 0.78; 95% CI, 0.68–0.90). In data analysis from multiple studies, liver metastases could be used as an independent prognostic risk factor, increasing the risk of death by 21% in lung cancer patients receiving ICI treatment compared with those without liver metastases (censored OS HR, 1.21; 95% CI, 1.17–1.27; *p* < 0.0001) [39].

## Data Availability

The raw data supporting the conclusions of this article will be made available by the authors, without undue reservation.
